# Dual-mechanism small bowel obstruction from Meckel diverticulum in an 8-year-old: a case report

**DOI:** 10.1093/jscr/rjag393

**Published:** 2026-05-24

**Authors:** Omar Taher, Zachary Ta, Abdelaziz Farhat

**Affiliations:** Noorda College of Osteopathic Medicine, Provo, UT 84604, United States; Pediatrix Medical Group, Timpanogos Regional Hospital, Orem, UT 84057, United States; Pediatrix Medical Group, Timpanogos Regional Hospital, Orem, UT 84057, United States

**Keywords:** Meckel diverticulum, small bowel obstruction, omphalomesenteric duct, postoperative complications, intestinal adhesions, laparoscopy

## Abstract

We report a dual-etiology obstruction in an 8-year-old boy: congenital closed-loop small bowel obstruction (SBO) from a volvulized Meckel diverticulum tethered by an omphalomesenteric duct remnant, followed by early postoperative focal SBO from an omental band. He presented after one week of abdominal pain with worsening and non-bloody, non-bilious emesis. Contrast-enhanced computed tomography showed distal SBO with a mesenteric swirl. Laparoscopy confirmed a necrotic Meckel diverticulum with volvulized distal ileum; the tethering band was divided and segmental ileal resection with anastomosis performed. After transient return of bowel function, he developed recurrent distention and bilious emesis; imaging showed a discrete transition point near the anastomosis with decompressed distal bowel and colon. Symptoms persisted despite decompression and parenteral nutrition. Repeat laparoscopy revealed a single omental band proximal to the anastomosis; adhesiolysis resolved obstruction. Clinical lesson: early focal postoperative SBO with a discrete transition point may warrant prompt minimally invasive re-exploration.

## Introduction

Meckel diverticulum, a remnant of the omphalomesenteric duct, is the most common congenital anomaly of the gastrointestinal tract [1, 2]. Children may present with bleeding, inflammation, or small bowel obstruction (SBO) [1–3]. SBO most often results from intussusception or a fibrous omphalomesenteric/mesodiverticular band [2–6]. Early postoperative mechanical SBO after Meckel resection in children appears uncommon and is less frequently described than primary presentations [7–9]. We describe an 8-year-old with closed-loop SBO from a volvulized Meckel diverticulum tethered by an omphalomesenteric remnant who subsequently developed an early postoperative focal SBO from a single omental band, managed with re-laparoscopy. We emphasize practical clinical and imaging features that favored early mechanical obstruction over ileus and supported timely minimally invasive re-exploration when progress stalled.

## Case report

**Figure 1:**
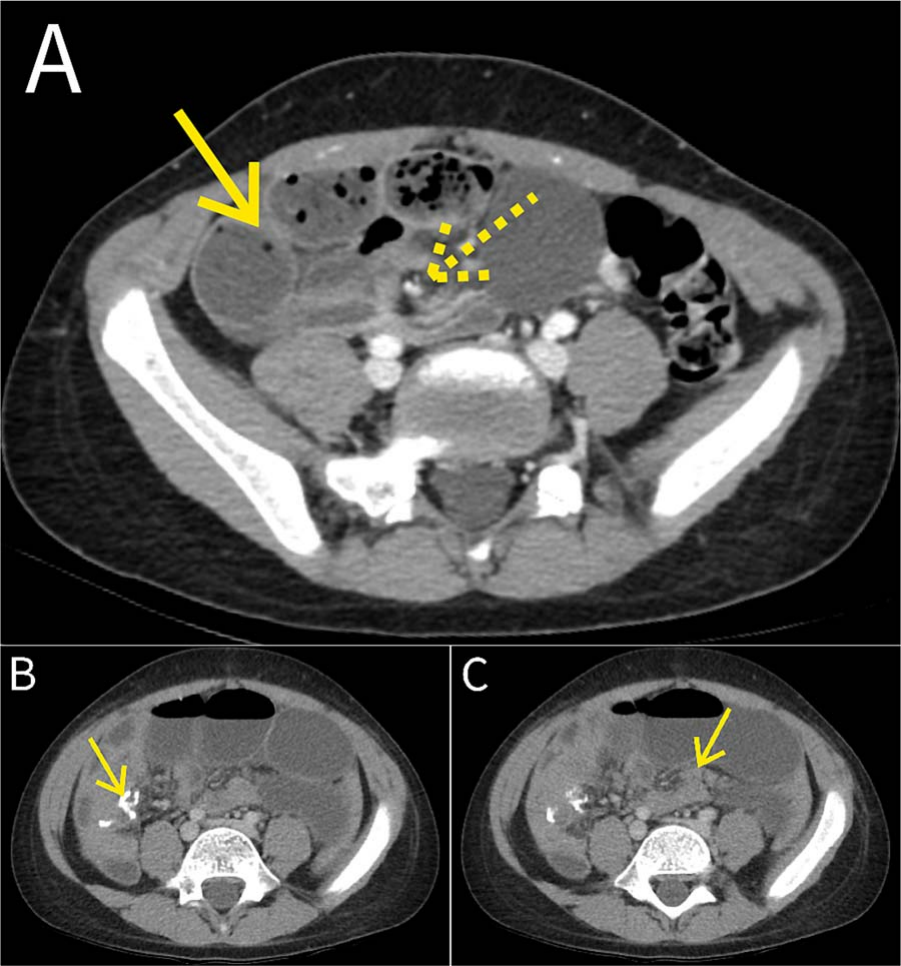
CT findings. (A) Contrast-enhanced CT of the abdomen and pelvis at the outside hospital demonstrating distended distal small-bowel loops with faecalization and mesenteric swirl, concerning for high-grade small bowel obstruction (SBO). The dashed arrow indicates the mesenteric swirl, and the solid arrow indicates a dilated distal small-bowel loop. The appendix is not dilated. (B) Postoperative contrast-enhanced CT obtained after recurrence of obstructive symptoms demonstrating the stapled small-bowel anastomosis (arrows). (C) Postoperative contrast-enhanced CT demonstrating a transition point in the right abdomen a few centimeters medial to the anastomosis (arrows), with marked proximal small-bowel dilatation and decompressed distal small bowel and colon, consistent with high-grade mechanical SBO. No abscess or anastomotic leak is identified.

An 8-year-old boy with no prior abdominal surgery presented after one week of progressive abdominal pain, worsening over 2–3 days, and several episodes of non-bloody, non-bilious emesis. At an outside hospital earlier that day, computed tomography (CT) with intravenous contrast demonstrated distended distal small-bowel loops measuring up to 2.8 cm with fecalization and mesenteric swirling, concerning for high-grade closed-loop SBO (Fig. 1A). He was transferred the same evening to our institution for pediatric surgical management.

On arrival, he was stable with mild abdominal distention and lower abdominal tenderness. Laboratory evaluation showed mild leukocytosis without metabolic derangement. Given concern for closed-loop obstruction, he underwent diagnostic laparoscopy within approximately 3–4 hours of arrival.

**Figure 2:**
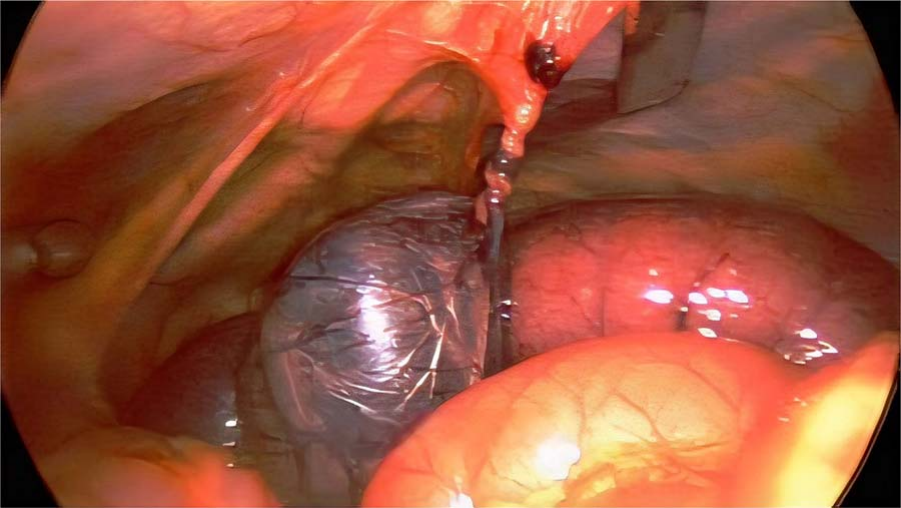
Laparoscopic findings at the index operation. Ischemic/necrotic, volvulized Meckel diverticulum with adjacent distal ileum, tethered to the anterior abdominal wall by a fibrous omphalomesenteric duct remnant (band), consistent with a closed-loop small bowel obstruction.

Laparoscopy revealed volvulized distal ileum wrapped around a nonviable Meckel diverticulum tethered to the anterior abdominal wall by a fibrous omphalomesenteric duct remnant, creating a closed-loop obstruction (Fig. 2). The band was divided and the bowel detorsed; adjacent ileum reperfused and no perforation or gross contamination was identified. The diverticulum remained ischemic, so a short segment ileal resection with stapled side-to-side anastomosis was performed. No additional obstructing bands or malrotation were identified. Pathology confirmed Meckel diverticulum with gastric heterotopia and negative margins; gross length was 5.5 cm.

Postoperatively, he was admitted to the pediatric intensive care unit (PICU) for close monitoring given the preoperative closed-loop obstruction and concern for ischemia and fluid shifts. He initially improved with flatus and stool. By postoperative days (POD) 4–5, recurrent distention, bilious emesis, and decreased oral intake represented new deterioration after transient recovery rather than routine follow-up. Because radiographs did not adequately distinguish ileus from focal mechanical SBO or an anastomotic complication, repeat CT was obtained, demonstrating a discrete transition point a few centimeters medial to the anastomosis (Fig. 1B and C), with marked proximal dilation and decompressed distal bowel and colon. No abscess or anastomotic leak was identified.

Because he had received minimal enteral nutrition for >1 week, total parenteral nutrition (TPN) via a peripherally inserted central catheter (PICC) was started on POD 5. When symptoms and imaging failed to improve, a nasogastric tube (NGT) to low intermittent suction was placed on POD 6 and he was kept nil per os. Despite bowel rest and decompression, he had persistent distention, bilious NGT output, and no passage of stool or flatus. Repeat radiographs remained concerning for high-grade obstruction with minimal colonic gas. CT suggested a single transition point, and bowel caliber was manageable for minimally invasive visualization. Given the focal transition point, lack of clinical improvement, and absence of peritonitis, the team elected repeat laparoscopy to confirm the diagnosis and perform targeted adhesiolysis while minimizing morbidity.

**Figure 3:**
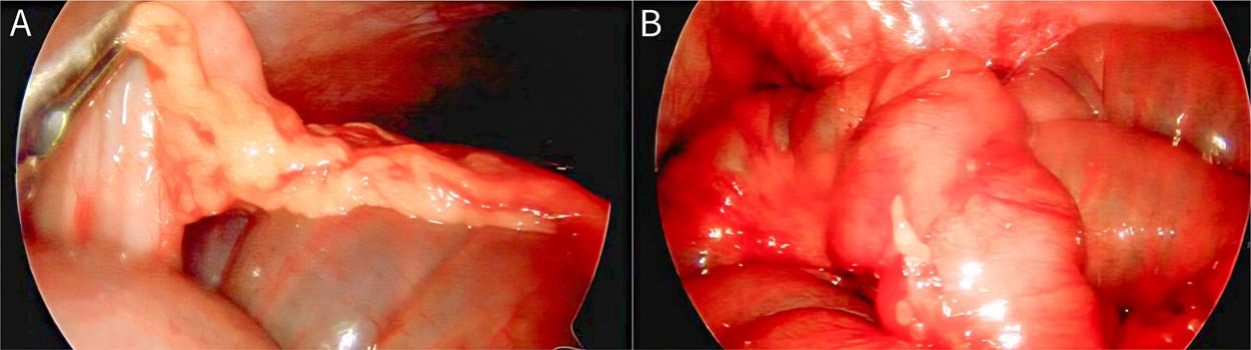
Laparoscopic findings at re-exploration on POD 7. (A) Omental adhesion band adherent to the ileum just proximal to the anastomosis, creating a constricting band and discrete transition point consistent with mechanical small bowel obstruction. (B) Side-to-side small bowel–small bowel anastomosis after adhesiolysis, appearing intact and patent; no further resection was required.

On POD 7, repeat laparoscopy was performed through the prior port sites. The small bowel was examined, revealing an isolated omental adhesion to the ileum just proximal to the anastomosis, forming a constricting band at the transition point (Fig. 3). The omental band and minor adhesions near the anastomosis were divided. The anastomosis appeared well perfused, intact, and widely patent, with gas and enteric contents observed to pass across it and distend the distal ileum toward the ileocecal valve, confirming it was not the source of obstruction; no abscess or leak was identified and no further resection was required.

**Figure 4:**
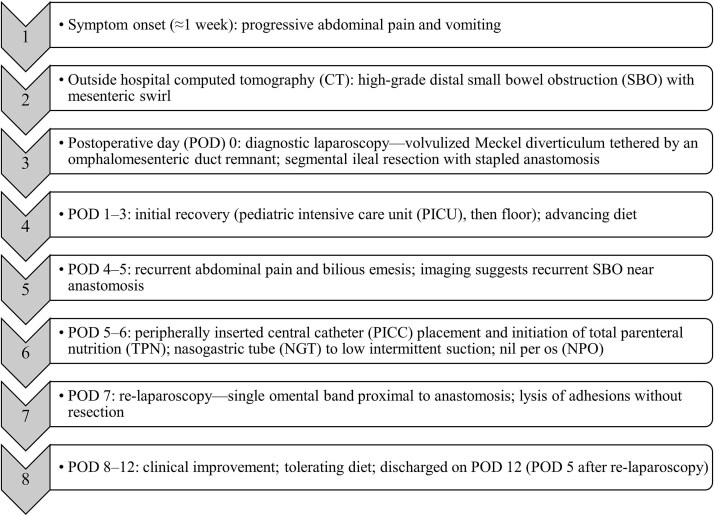
Timeline of the patient’s presentation, operative management, and postoperative course shown by POD relative to the index operation. Abbreviations: Computed tomography (CT); small bowel obstruction (SBO); pediatric intensive care unit (PICU); peripherally inserted central catheter (PICC); total parenteral nutrition (TPN); nasogastric tube (NGT); nil per os (NPO).

He recovered with resolution of distention, return of bowel function, and advancement to a regular diet. TPN and the PICC were discontinued, and he was discharged home in good condition 5 days after re-laparoscopy (POD 12). He remained asymptomatic with no recurrent obstructive episodes. A timeline is shown in Fig. 4.

## Discussion

Meckel diverticulum occurs in ~2% of the population [1, 2]. In children, SBO can result from intussusception, volvulus around a fibrous band, internal hernia, or adhesions after resection [2–6]. This case is notable for dual-mechanism obstruction: congenital closed-loop SBO from an omphalomesenteric remnant, followed by early postoperative recurrence from a single omental band proximal to an intact anastomosis.

Early postoperative mechanical SBO is less common than postoperative ileus, but persistent bilious emesis, failure to advance diet, and imaging demonstrating a discrete transition point with decompressed distal bowel and colon should raise concern for a mechanical process [7–9]. Pediatric series and reviews note that early postoperative SBO often has a single correctable mechanical etiology—such as an adhesive band, internal hernia, or focal kink—identified at re-exploration [7–10].

Although pediatric radiation exposure must be minimized, the postoperative CT was obtained for a management-changing question rather than surveillance: recurrent bilious emesis and distention after transient recovery raised concern for focal mechanical SBO or an anastomotic complication rather than ileus alone. The resulting transition point supported early re-laparoscopy rather than continued treatment as ileus.

In our patient, timely minimally invasive re-exploration was diagnostic and therapeutic, allowing full small-bowel evaluation, division of the omental band, and confirmation that the anastomosis was viable and patent [7–10]. This supports early operative reassessment when clinical progress stalls, particularly when imaging suggests a focal transition point.

In children with a ‘virgin abdomen’ and imaging concerning for closed-loop SBO, Meckel diverticulum with associated bands should remain high on the differential [2–6]. Although meticulous technique and minimizing tissue trauma may reduce postoperative adhesions, not all adhesions are avoidable [7–10]. This report follows the Surgical CAse REport guidelines [11].
